# Supplementary material for: Control of transcription elongation and DNA repair by alarmone ppGpp
Source: Nat Struct Mol Biol. 2023 Mar 30;30(5):600–7. doi: 10.1038/s41594-023-00948-2 (PMC10191844; doi:10.1038/s41594-023-00948-2)
Supplement: Supplementary file 1 — Supplementary text. [file 41594_2023_948_MOESM1_ESM.pdf]

nature structural & molecular biology

Article

https://doi.org/10.1038/s41594-023-00948-2

Control of transcription elongation and DNA repair by alarmone ppGpp

In the format provided by the authors and unedited

Supplementary Text

Comparison of the ppGpp binding to Site 1 in the elongation and initiation complexes.

Comparison with 4JK1: This 3.9 Å X-ray crystal structure[20] correctly identified ppGpp-binding site between β′ and ω subunits of RNAP but incorrectly placed nucleobase in the density map of the diphosphate.

Comparison with 4JKR: Compared to the current structure of the ppGpp-EC and other structures of the initiation complexes, this published X-ray crystal structure[16] places ppGpp with the nucleoside face flipped, resulting in no base specific interaction. Then, the 5′ and 3′ diphosphates occupy the position of the 3′ and 5′ diphosphates in the ppGpp-EC structure, respectively. In this conformation, the majority of the backbone-specific interactions are formed with the 5′ diphosphate, involving main chain functionalities of ω Ala2, Arg3, and Val4, and a guanidinium group of β′ Lys615 (Extended Data Fig. 4c). In contrast, each phosphate of the 3′ diphosphate forms only one hydrogen bond with the side chains of ω Arg3 and Arg52. Thus, this structure cannot explain efficient discrimination of ppGpp against GTP and GDP as well as RNAs containing these nucleotides on the 5′ end. In addition, the side chain of β′ Arg362 is moved away from the nucleobase of ppGpp and does not form cation-π-interactions (Extended Data Fig. 4c, bottom). Instead, Arg362 recognizes 2′ OH of the ribose. β′ His364 is positioned perpendicular to the ppGpp nucleobase and has potential for C- H-π bond. The nucleobase is also shifted away from β′ Ile619 so that it forms only weak VDW interactions.

Comparison with 5VSW: This 4.29 Å X-ray crystal structure[17] contains two assemblies of the RNAP-RpoD-DksA-ppGpp complex in the asymmetric unit. As for the 4JKR structure, the low resolution of the electron density maps precluded visualization of the protein side chains and practically no contacts between ppGpp in Site 1 and RNAP could be confirmed unambiguously. Nevertheless, ppGpp molecule was placed in the orientation identical to the current structure. Despite the lack of the density map for some amino acids and unresolved maps for other residues, several important intermolecular interactions, observed in the current structure, were also captured in the 5VSW structure. Structural models for chains D and E suggested binding of the 3′ phosphates by Ala2, Arg3 and Lys615, recognition of the Watson-Crick edge of guanosine by Asp622, cation-π interactions with Arg362, and VDW interactions with Ile619. Most but not all these contacts were also observed in the second set of molecules of the asymmetric unit.

Comparison with 7KHI and 7KHE: These 3.62 and 3.58 Å cryo-EM structures[21] contain the RNAP-RpoD-DksA-ppGpp complex initiating transcription on the *rrnBP1* promoter. The EM maps are of very good quality to unambiguously place most of the amino acid side chains in Site 1 (Extended Data Fig. 4d, top). The ppGpp binding sites are similar in the structures although conformations of the 5′ phosphates in ppGpp differ. These structures revealed many ppGpp-RNAP interactions similar to ones in the current ppGpp-EC structure. Specifically, they show similar recognition of the nucleobase by Arg362, Ile629, Asp622 and Gln623 and the 5′ Pβ phosphate by Arg3 and Arg417 (Extended Data Fig. 4d). Binding of the 3′ Pβ by Ala2, Arg3 and Lys615 also features many similarities with the EC-ppGpp structure.
