# Supplementary material for: Control of transcription elongation and DNA repair by alarmone ppGpp
Source: Nat Struct Mol Biol. 2023 Mar 30;30(5):600–7. doi: 10.1038/s41594-023-00948-2 (PMC10191844; doi:10.1038/s41594-023-00948-2)
Supplement: Supplementary file 2 — Reporting Summary [file 41594_2023_948_MOESM2_ESM.pdf]

nature portfolio

Corresponding author(s): Evgeny Nudler
Alexander Serganov

Last updated by author(s): Jan 31, 2023

Reporting Summary

Nature Portfolio wishes to improve the reproducibility of the work that we publish. This form provides structure for consistency and transparency in reporting. For further information on Nature Portfolio policies, see our Editorial Policies and the Editorial Policy Checklist.

Statistics

For all statistical analyses, confirm that the following items are present in the figure legend, table legend, main text, or Methods section.

n/a	Confirmed	
☐	☒	The exact sample size (*n*) for each experimental group/condition, given as a discrete number and unit of measurement
☐	☒	A statement on whether measurements were taken from distinct samples or whether the same sample was measured repeatedly
☐	☒	The statistical test(s) used AND whether they are one- or two-sided<br>*Only common tests should be described solely by name; describe more complex techniques in the Methods section.*
☒	☐	A description of all covariates tested
☐	☒	A description of any assumptions or corrections, such as tests of normality and adjustment for multiple comparisons
☐	☒	A full description of the statistical parameters including central tendency (e.g. means) or other basic estimates (e.g. regression coefficient) AND variation (e.g. standard deviation) or associated estimates of uncertainty (e.g. confidence intervals)
☐	☒	For null hypothesis testing, the test statistic (e.g. *F*, *t*, *r*) with confidence intervals, effect sizes, degrees of freedom and *P* value noted<br>*Give P values as exact values whenever suitable.*
☒	☐	For Bayesian analysis, information on the choice of priors and Markov chain Monte Carlo settings
☒	☐	For hierarchical and complex designs, identification of the appropriate level for tests and full reporting of outcomes
☒	☐	Estimates of effect sizes (e.g. Cohen's *d*, Pearson's *r*), indicating how they were calculated

Our web collection on statistics for biologists contains articles on many of the points above.

Software and code

Policy information about availability of computer code

Data collection	CryoEM: Leginon 3.5
Data analysis	CryoSPARC v 3.3.2<br>Phenix v 1.17.1<br>Coot v 0.8.9.2<br>PyMol v 2.5.4<br>Chimera v 1.15<br>GraphPad Prism v 8.4.3

For manuscripts utilizing custom algorithms or software that are central to the research but not yet described in published literature, software must be made available to editors and reviewers. We strongly encourage code deposition in a community repository (e.g. GitHub). See the Nature Portfolio guidelines for submitting code & software for further information.

Data

Policy information about availability of data

All manuscripts must include a data availability statement. This statement should provide the following information, where applicable:

- Accession codes, unique identifiers, or web links for publicly available datasets
- A description of any restrictions on data availability
- For clinical datasets or third party data, please ensure that the statement adheres to our policy

CryoEM maps and atomic coordinates are deposited in the Electron Microscopy Data Bank and Protein Data Bank, respectively. Accession codes are PDB 8FVR and

EMBD 29491 for EC and PDB 8FVW and EMDB 29494 for EC-ppGpp.All other data are available in the manuscript or supplementary materials. Requests for strains or plasmids will be fulfilled by the lead contact author, E.N., upon request.

Field-specific reporting

Please select the one below that is the best fit for your research. If you are not sure, read the appropriate sections before making your selection.

☒ Life sciences ☐ Behavioural & social sciences ☐ Ecological, evolutionary & environmental sciences

For a reference copy of the document with all sections, see nature.com/documents/nr-reporting-summary-flat.pdf

Life sciences study design

All studies must disclose on these points even when the disclosure is negative.

Sample size	No sample size calculation was performed. Independent experiments were conducted for reproducibility. The number of experiments were chosen based on the standards of the field (at least 2 biological replicates) and the total number of individual experiments depended on the reproducibility of the results.
Data exclusions	Particle images were excluded during cryoEM data processing following 2D classification based on lack of high-resolution detail following standard procedure for cryoEM structure determination.
Replication	Replication of experiments was successful. The number of independent biological experiments is indicated in Methods and Figure legends.
Randomization	Randomization was not used for this study as outcomes did not depend upon researcher bias.
Blinding	Blinding was not employed for this study as outcomes did not depend upon researcher bias. Replication of experiments showed good reproducibility.

Reporting for specific materials, systems and methods

We require information from authors about some types of materials, experimental systems and methods used in many studies. Here, indicate whether each material, system or method listed is relevant to your study. If you are not sure if a list item applies to your research, read the appropriate section before selecting a response.

Materials & experimental systems

n/a	Involved in the study
☒	☐ Antibodies
☒	☐ Eukaryotic cell lines
☒	☐ Palaeontology and archaeology
☒	☐ Animals and other organisms
☒	☐ Human research participants
☒	☐ Clinical data
☒	☐ Dual use research of concern

Methods

n/a	Involved in the study
☒	☐ ChIP-seq
☒	☐ Flow cytometry
☒	☐ MRI-based neuroimaging
